# *INTS6/DICE1 *inhibits growth of human androgen-independent prostate cancer cells by altering the cell cycle profile and Wnt signaling

**DOI:** 10.1186/1475-2867-9-28

**Published:** 2009-11-11

**Authors:** Stephanie Filleur, Jennifer Hirsch, Aline Wille, Margarete Schön, Christian Sell, Michael H Shearer, Thomas Nelius, Ilse Wieland

**Affiliations:** 1 Texas Tech University Health Sciences Center, Department of Urology, Lubbock, TX, USA; 2 Texas Tech University Health Sciences Center, Department of Microbiology and Immunology, Lubbock, TX, USA; 3 Institute for Human Genetics, Otto-von-Guericke University, Magdeburg, Germany; 4 Rudolf Virchow Center, DFG Research Center for Experimental Biomedicine and Department of Dermatology, University of Würzburg, Würzburg, Germany; 5 Department of Pathology, Drexel University College of Medicine, Philadelphia, PA, USA

## Abstract

**Background:**

The gene encoding integrator complex subunit 6 (*INTS6*), previously known as deleted in cancer cells 1 (*DICE1*, OMIM 604331) was found to be frequently affected by allelic deletion and promoter hypermethylation in prostate cancer specimens and cell lines. A missense mutation has been detected in prostate cancer cell line LNCaP. Together, these results suggest *INTS6/DICE1 *as a putative tumor suppressor gene in prostate cancer. In this study, we examined the growth inhibitory effects of *INTS6/DICE1 *on prostate cancer cells.

**Results:**

Markedly decreased *INTS6/DICE1 *mRNA levels were detected in prostate cancer cell lines LNCaP, DU145 and PC3 as well as CPTX1532 as compared to a cell line derived from normal prostate tissue, NPTX1532. Exogenous re-expression of *INTS6/DICE1 *cDNA in androgen-independent PC3 and DU145 cell lines substantially suppressed their ability to form colonies *in vitro*. This growth inhibition was not due to immediate induction of apoptosis. Rather, prostate cancer cells arrested in G1 phase of the cell cycle. Expression profiling of members of the Wnt signaling pathway revealed up-regulation of several genes including disheveled inhibitor CXXC finger 4 (*CXXC4*), frizzled homologue 7 (*FZD7*), transcription factor 7-like 1 (*TCF7L1*), and down-regulation of cyclin D1.

**Conclusion:**

These results show for the first time a link between *INTS6/DICE1 *function, cell cycle regulation and cell-cell communication involving members of the Wnt signaling pathway.

## Background

Prostate cancer is the most common malignancy and the second leading cause of cancer-related death in men from western countries. The American Cancer Society (ACS) estimated for 2009, 192,280 new cases of prostate cancer in the United States with approximately 27,360 cases ending in death (ACS, Cancer statistics, 2009). Although these numbers reflect the recent progress in prostate cancer therapy, they also reveal the demand for more effective therapeutic strategies.

Progression of prostate cancer to androgen-independence has been associated with multiple molecular mechanisms such as androgen receptor (AR) gene amplification, *AR *gene mutations resulting in AR hypersensitivity or change of AR specificity, involvement of coregulators, ligand independent activation of the AR, and involvement of tumor stem cells [1]. More recently, other androgen-independent mechanisms involving dysregulation of several cell-survival signaling pathways in androgen-independent prostate cancer have also been established. *Deleted In Cancer 1 *(*INTS6/DICE1*) gene (OMIM 604331) was identified to colocalize with the microsatellite marker *D13S284 *in 13q14.3, a region frequently affected by allelic deletion in many solid tumors including prostate cancer [2-5]. *INTS6/DICE1 *missense mutations have been previously detected in lung and prostate cancer cell lines NCI-H2126 and LNCaP respectively, and reduced *INTS6/DICE1 *expression appears to be associated with CpG promoter hypermethylation in lung and prostate cancer cells [3,5-7]. DICE1 is a 100 kD widely expressed and highly conserved nuclear protein with predicted protein motifs reminiscent of classical DEAD box helicases suggesting its involvement in important nuclear functions such as DNA repair, transcription or RNA splicing [2,3,8]. In agreement with its nuclear localization, human DICE1 was detected to be subunit 6 of the integrator complex (INTS6) involved in small nuclear RNA processing [9]. This integrator complex is competent for transcription and may be recruited to the promoter of RNA polymerase II-dependent genes. Recently, *INTS6/DICE1 *has been identified as one of the target genes of CCAAT enhancer binding protein delta (C/EBPδ) which is highly expressed in growth arrested, contact inhibited mammary epithelial cells [10]. *INTS6*/*DICE1 *is also subject to regulation by the tumor suppressor CDC73 that is mutually inactivated in hereditary and sporadic parathyroid tumors [11]. In mouse, the *INTS6*/*DICE1 *homologue interfered with the response to insulin-like growth factor 1 (IGF-1), and mouse and human *DICE1 *cDNA suppressed anchorage-independent growth of transformed mouse cells [12,13]. These results suggest that *INTS6/DICE1 *is a tumor suppressor gene and emphasize the need to better characterize its function.

## Results

### INTS6/DICE1 mRNA expression is down-regulated in prostate cancer cells

**Figure 1:**
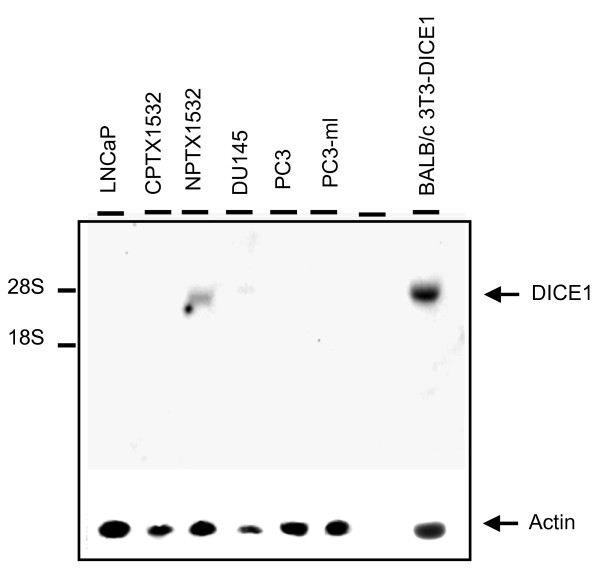
**Northern blot of prostate cancer cell lines**. LNCaP, DU145, PC3, PC3-ml and CPTX1532 and NPTX1532 (cells derived from cancer and normal prostate tissue) were analyzed using a full length *DICE1 *cDNA as a hybridization probe. BALB/c 3T3 cells transduced by full length *DICE1 *cDNA were used as a positive control. The position of *DICE1 *mRNA is indicated by an arrow on the right side and the positions of the 18S and 28S RNA are indicated by the hash marks on the left side. In the lower panel the same blot was subsequently probed for beta actin as a control for the relative amount of RNA in each sample. Note that two distinct batches of mRNA were collected from prostatic cell lines and gave similar expression profile for *DICE1 *mRNA.

In order to reinforce *DICE1 *tumor suppressor function in prostate cancer, we analyzed its expression in different human androgen-dependent/-sensitive (LNCaP), androgen-independent/-insensitive (DU145, PC3, PC3-ml); and prostate cell lines isolated from prostatic primary tumor (paired normal/tumor 1532NPTX/1532CPTX cell lines) [14]. Northern blot showed that *DICE-1 *mRNA in all the prostate tumor cell lines tested is strongly down-regulated or undetectable compared to normal immortalized prostate 1532NPTX cells and BALB/c3T3 mouse cells transfected with the full length *DICE1 *cDNA (Figure 1).

### Exogenous expression of *DICE1 *inhibits the clonogenic formation capacity of androgen-independent cell lines through an apoptosis-independent pathway

**Figure 2:**
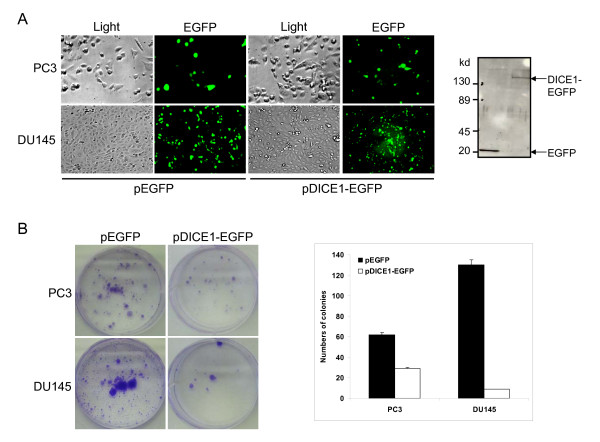
**Inhibition of colony formation of PC3 and DU145 prostate cancer cells by pDICE1-EGFP fusion construct**. PC3 and DU145 cells were transiently transfected with the pEGFP or pDICE1-EGFP fusion constructs. A. Equivalent transfection efficiencies (30-40%) at 48 hours post-transfection were verified by reporting EGFP *in situ *fluorescence on the total number of cells (regular light) using an inverted microscope (left). Expression of constructs was analyzed in PC3 cells by Western blotting of EGFP and DICE1-EGFP fusion protein using an anti-GFP monoclonal antibody (right). Sizes in kilodalton (kD) are indicated on the left. B. After transfection, prostate cancer cells were selected over 2-3 weeks in the presence of G418 antibiotic and stained with 0.5% crystal violet in methanol (left). Quantitative analysis of inhibition of the colony formation in PC3 and DU145 cells transfected with pEGFP and pDICE1-EGFP plasmid (right).

*DICE1 *function in prostate cancer was studied by exogenously re-expressing *DICE1 *cDNA in the prostate tumor cell lines PC3 and DU145. This experiment was performed by transfecting the cancer cells with the pDICE1-EGFP fusion construct or pEGFP control expression plasmid (Figure 2a, left). Synthesis of a DICE1-EGFP fusion protein in PC3 (Figure 2a, right) and DU145 (data not shown) transfected cells was shown by a band around 150 kD by Western blot analysis. This size is slightly larger than the calculated size (expected ~125 kD). Similarly, a slightly larger than expected size band was previously found for human endogenous DICE1 protein functioning as integrator subunit 6 of the integrator multiprotein complex [9].

Transfected cells were then tested for their ability to form colonies *in vitro *in G418 supplemented selective medium. Interestingly, we show that the re-expression of *DICE1 *inhibits colony formation of PC3 and DU145 cells by 50% and 90% respectively when compared to the control pEGFP-transfected cells (Figure 2b). With the androgen-dependent cell line LNCaP, previously shown to harbor missense mutation D546G in the *DICE1 *gene [6], we obtained inconsistent results concerning the growth-inhibitory responses to DICE1-EGFP (data not shown).

To determine if *DICE1 *suppresses prostate cancer cell growth by inducing apoptosis, we analyzed its capacity to stimulate genomic DNA fragmentation in PC3 cells. PC3 cells were transiently transfected with pDICE1-EGFP or pEGFP plasmid. Subsequently, genomic DNA was extracted after 24 and 48 hours, quantified and loaded on an agarose gel. We could not detect any DNA fragmentation in PC3 transfected cells as compared to the apoptotic positive control cells (data not shown). These results were validated by loading higher amounts of genomic DNA (up to 10 μg, data not shown). In our understanding, the absence of an apoptotic signal could not be explained by a lack of sensitivity of the present assay. Indeed, we estimated approximately 30% transfection efficiency in this experiment, which is an adequate ratio to visualize DNA fragmentation. Additionally, we tested the capacity of *DICE1 *to induce necrosis in androgen-independent cells. As expected, this analysis performed by propidium iodide (PI) uptake assay on prostate cancer cells did not show any significant effect of *DICE1 *expression on the percentage of PI-positive cells (data not shown). In conclusion, our results suggest that *DICE1 *is inhibiting prostate cancer cell growth through an apoptosis- and necrosis-independent pathway.

### *DICE1 *expression induces G1 arrest in androgen-independent cell lines

**Figure 3:**
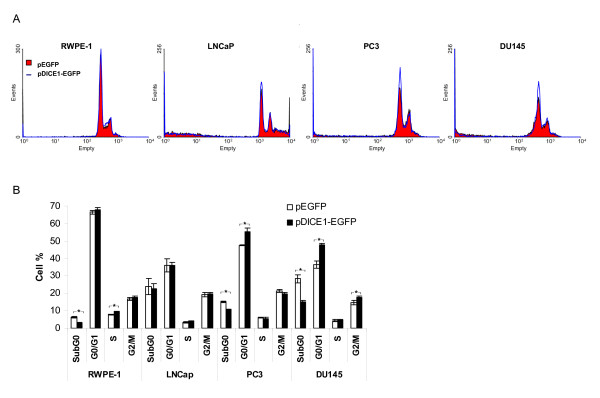
**Cell cycle distribution in response to *INTS6/DICE1 *re-expression**. A. Normal prostate (RWPE-1) and cancer prostate (LNCaP, PC3 and DU145) cells were transfected with pEGFP or pDICE1-EGFP expression plasmid, stained with propidium iodide and the DNA content was analyzed by flow cytometry. B. Quantification of flow cytometry analysis of DNA content. Results are represented as the average values +/- SD calculated from three separate experiments. *: P < 0.05. Note the increase in the G1 cell population in PC3 and DU145 cells expressing pDICE1-EGFP compared to pEGFP cancer cells.

To further understand the mechanisms involved in *DICE1 *growth inhibitory function, we examined the capacity of *DICE1 *to modify the cell cycle distribution of normal prostate cells RWPE-1 and prostate cancer cells LNCaP, PC3 and DU145. Similar to results obtained with normal RWPE-1 cells, re-expression of *DICE1 *in PC3 or DU145 cells induced a 30-50% cell reduction in the sub-G0 phase (15.1 +/- 0.52 for pEGFP-PC3 versus 10.4 +/- 0.54 for pDICE1-EGFP-PC3 and 28.4 +/- 2.21 for pEGFP-DU145 versus 14.9 -/+ 1.18 for pDICE1-EGFP-DU145), validating our hypothesis of an apoptosis-independent mechanism (Figure 3a-b). Likewise, the percentage of PC3 and DU145 cells in G0/G1 was increased about 20-30% following DICE1 re-expression suggesting a block in the G1-phase of the cell cycle (Figure 3a; 47.5 +/- 0.23 for pEGFP-PC3 versus 55.3 +/- 2.26 for pDICE1-EGFP-PC3 and 36.4 +/- 2.24 for pEGFP-DU145 versus 47.6 +/- 1.0 for pDICE1-EGFP-DU145). DU145 cells re-expressing DICE1 showed an additional 20% increase in cell number in G2/M phase emphasizing that DICE-1 could regulate the cell cycle at different levels. In agreement with the results obtained in the colony formation assay, the re-expression of DICE1 in LNCaP cell line did not present any significant effect on their cell cycle distribution.

### Changes in the Wnt signaling pathway in response to *DICE1*

**Figure 4:**
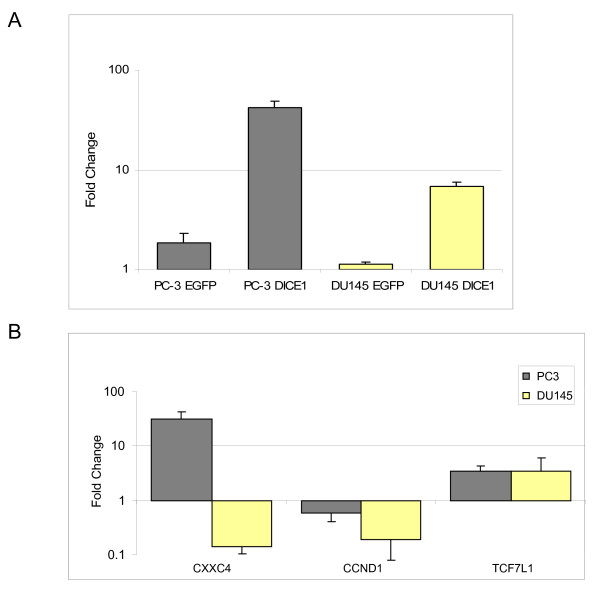
**Real-time PCR of *INTS6/DICE1 *responsive genes**. A. Up-regulation of *INTS6/DICE1 *expression in PC3 and DU145 cells 48 hours post-transfection with DICE1-EGFP fusion construct (DICE1) or EGFP vector (EGFP) as control. B. Selected *INTS6/DICE1 *responsive genes of the Wnt signaling pathway, CXX finger 4 (*CXXC4*), cyclin D1 (*CCND1*) and transcription factor 7-like 1 (*TCFL1*) monitored 48 hours after exogenous *DICE1 *expression in PC3 and DU145 cells. The fold change in gene expression was determined by the comparative C(T) method using G3PDH as reference.

As previously described, inhibition of the Wnt pathway in PC3 cells resulted in decreased colony formation in soft agar and *in vivo *tumor growth [15]. Therefore, we analyzed the expression of genes related to this pathway by PCR array. PC3 cells containing the exogenous pDICE1-EGFP fusion construct showed differences in the expression of several genes when compared with pEGFP-transfected PC3 cells indicating regulation of Wnt signaling in response to exogenous *DICE1 *expression (Table 1). In particular, genes like CXXC finger 4 (*CXXC4*), frizzled homolog 7 (*FZD7*) and transcription factor 7-like 1 (*TCF7L1*) showed an up-regulation of more than 3- to 7-fold, whereas cyclin D1 and transcription factor 7 (*TCF7*) were clearly down-regulated (3.8 and 3.6 fold, respectively; Table 1). To confirm the PCR array results, we repeated the expression analysis by quantitative real-time PCR for selected genes *CXXC4*, *TCF7L1 *and *CCND1 *in *DICE1*- and control-transfected PC3 and DU145 cells. The data showed a clear 3.5 fold up-regulation of *TCF7L1 *in PC3 and DU145 cells in response to *DICE1*, whereas *CCND1 *was down-regulated (1.7 and 5.3 fold, respectively). In the case of *CXXC4*, *DICE1*-transfected PC3 cells showed a more than 30-fold up-regulation, whereas a 7-fold down-regulation was observed in DU145 cells (Figure 4).

**Table 1:** Expression profiling of Wnt signaling pathway in *INTS6/DICE1 *transfected PC3 cells

Gene Symbol	Description	GenBank	FC
Upregulated genes^a^			
AXIN1	Axin 1	NM_003502	2.14
CTBP2	C-terminal binding protein 2	NM_022802	2.14
CXXC4	CXXC finger 4	NM_025212	7.5
FZD7	Frizzled homolog 7 (Drosophila)	NM_003507	4.0
JUN	Jun Oncogen	NM_002228	2.8
MYC	V-myc myelocytomatosis viral oncogen homolog (avian)	NM_002467	4.0
SLC9A3R1	Solute carrier family 9 (sodium/hydrogen exchanger)	NM_004252	2.64
T	T, brachyury homolog (mouse)	NM_003181	2.3
TCF7L1	Transcription factor 7-like 1 (T-cell specific, HMG-box)	NM_031283	3.24
TLE	Transcucin-like enhancer of split (E(sp1)homolog, Drosophila	NM_005077	2.14
WISP1	WNT1 inducible signalling pathway protein	NM_003882	3.24
WNT3	Wingless-type MMTV integration site family, member 3	NM_030753	2.3
WNT5B	Wingless-type MMTV integration site family, member 5B	NM_032642	3.5
Downregulated genes^b^			
APC	Adenomatosis polyposis coli	NM_000038	-2.5
BTRC	Beta-transducin repeat containing	NM_033637	-2.7
CCND1	Cyclin D1	NM_053056	-3.8
FBXW2	F-box and WD repeat domain containing 2	NM_012164	-3.3
FZD6	Frizzled homolog 6 (Drosophila)	NM_003506	-2.5
SENP2	SUMO1/sentrin/SMT3 specific peptidase 2	NM_021627	-2.3
TCF7	Transcription factor 7 (T-cell specific, HMG-box)	NM_003202	-3.6

a: FC, fold change >2, b: FC, fold change <0,5

## Discussion

Previous studies provided functional evidence that the *INTS6/DICE1 *gene acts as a tumor suppressor gene [13]. Regarding prostatic tumorigenesis, *INTS6/DICE1 *expression is down-regulated in multiple prostate cancer cell lines as compared to normal prostate cells, and its exogenous re-expression in cancer cells leads to inhibition of their capacity to form colonies *in vitro*. However, few results exist on the molecular mechanisms involved in *INTS6/DICE1 *growth-inhibitory function. In a mouse tissue culture model with IGF-IR transformed Balb/c 3T3 cells, it has been shown that mouse and human *DICE1 *cDNA inhibit anchorage-independent growth [12,13]. These results suggested a link between the IGF-IR signaling system and DICE1 function. Anchorage-independent growth, suppression of apoptosis, cell migration, invasion and metastasis are particularly abolished in mouse tumor cells by mutational changes of tyrosine residues 1250 and 1251 positioned outside the kinase domain of IGF-IR [16-18]. In human prostate cancer cells, blockade of IGF-IR expression by antisense cRNA inhibits proliferation and invasion and leads to an enhanced rate of spontaneous apoptosis [19]. However, apoptotic death was not observed when investigating the capacity of exogenous *INTS6/DICE1 *to induce the fragmentation of genomic DNA in prostate cancer cells. This suggested that DICE1 protein inhibits clonogenic cancer cell growth by bypassing an immediate apoptotic response. In agreement with this hypothesis, we were able to show that *DICE1 *re-expression in androgen-independent prostate cancer cells induced cell arrest in the G1 phase of the cell cycle identifying a molecular mechanism by which *DICE1 *could limit prostate cancer cell growth.

*INTS6/DICE1 *has been proposed to be a distant member of the DEAD box containing helicase superfamily II [3,8]. In this context, DICE1 protein has been recently identified as subunit 6 (INTS6) of the multi-protein Integrator complex involved in RNAPII-dependent transcription and processing of small nuclear RNA [9]. It now appears that modification in the expression level of *INTS6/DICE1 *could alter multi-protein complexes and consequently the gene expression profile in these cells. In prostate cancer with down-regulated *INTS6/DICE1 *expression, its exogenous expression may result in reassembly of DICE1 containing multi-protein complexes thus affecting distinct signaling pathways. Both IGF-1 receptor and Wnt signaling are fundamental pathways in tissue and organ development. Cross-talk between IGF-IR and Wnt signaling has been previously recognized during epithelium to mesenchymal transition as well as in Wnt-1/PTEN double transgenic mice [20,21]. A possible involvement of *INTS6/DICE1 *in growth arrest induction by serum and growth factor withdrawal and contact inhibition may also be inferred from the observation that *INTS6/DICE1 *is one of the primary target genes of C/EAPδ [10]. In fact, aberrantly activated Wnt signaling has also been implicated in prostate tumorigenesis and inhibition of the Wnt pathway in PC3 cells resulted in decreased colony formation in soft agar and *in vivo *tumor growth [22].

## Conclusion

The results obtained in this study link DICE1 function to fundamental pathways involved in cell cycle regulation and cell-cell communication. Understanding DICE1 modes of action as it relates specifically to its regulatory properties on the Wnt signaling pathway will provide novel insights in support of a role for DICE1 protein in prostate cancer progression and may potentially lead to development of improved therapeutic approaches to prostate cancer.

## Methods

### Cell Lines

Human immortalized normal prostate cells RWPE-1 (American Type Culture Collection; ATCC) were grown in keratinocyte serum-free medium (Invitrogen, Carlsbad, CA) supplemented with 25 μg/ml bovine pituitary extract and 5 ng/ml epidermal growth factor. Human prostate cancer cells LNCaP, DU145, PC3 and PC3-ml were grown in RPMI 1640 medium supplemented with 10% fetal calf serum (HyClone, Perbio Science, Erembodegem-Aalst, Belgium) and 1% penicillin/streptomycin mix (Sigma, Taufkirchen, Germany). Paired cancer/normal human prostate cell lines, CPTX1532 and NPTX1532, were generated from patient undergoing radical prostatectomy and established by immortalization after micro-dissection of primary tumor cells and adjacent normal tissue respectively [14]. NPTX1532 and CPTX1532 cell lines were cultured in keratinocyte serum-free medium, 25 μg/ml bovine pituitary extract, 5 ng/ml epidermal growth factor, 2 mM L-Glutamine, 10 mM HEPES, 50 ng/ml gentamicin sulphate (all from Invitrogen, Carlsbad, CA), 5% heat inactivated fetal bovin serum (BioWhitacker, Rockville, MD), 2.5% penicillin/streptomycin mix (CellGro) and 0.5 μg/ml fungizone (CellGro). For transfection, prostate cells (1 × 10^5^) were seeded in 35 mm culture dish and incubated overnight at 37°C. The cells were then transfected for 4 hours with 4 μg pDICE1-EGFP expression plasmid [13] or pEGFP control plasmid (Clontech) by using the CLONfectin kit (Clontech) following the recommended protocol. The transfection efficiencies were evaluated by counting the EGFP-positive cells under an inverted fluorescent microscope (Axiovert 25CFL, Zeiss, Göttingen, Germany), and reporting this number to the total number of cells (regular light).

### RNA isolation and Northern Blot analysis

BALB/c3T3 mouse cells were infected with retroviral particles containing either the full length *DICE1 *cDNA or no insert. The retroviral packaging line Phoenix (provided by Dr. Gary Nolan at Stanford University) was used for viral transduction studies [23]. Cells were selected in 1 μg/ml puromycin for 3 days. Total RNA was isolated following the guanidinium isothiocyanate method of Chomczynski and Sacchi [24]. Northern blot analysis was carried out using 10 mg of total RNA in glyoxal agarose gels. Size fractionated RNA was transferred to nylon filters electrophoretically in 1× Tris Acetate EDTA buffer (TAE). Probes were labeled by random priming and hybridization was carried out using a solution of 7% SDS, 0.25 M Na_3_PO_4_, 5.6 mM Na_4_P_2_PO_7_, 2 mM EDTA. Washing was carried out under standard conditions.

### Colony formation assay

After transfection, prostate cells were incubated over 2-3 weeks in the presence of G418 antibiotic (Sigma) to allow colonies to develop. At the endpoint of the experiment, the medium was removed, the colonies washed in PBS and stained with 0.5% crystal violet (Sigma) in methanol. Each colony formation assay was carried out in triplicate and repeated at least three times. The working concentration of G418 (380 μg/ml for PC3 and 240 μg/ml for DU145 cells) was defined as the lowest dose of antibiotic that kills 100% of non-transfected cells in 5-7 days from the start of G418 selection.

### Western blot analysis

Cell extracts of EGFP and DICE1-EGFP transfected cells were produced by RIPA lysis and separated by SDS-polyacrylamide gel electrophoresis (SDS-PAGE) according to Harlow and Lane [25]. The gel was blotted on to a PVDF transfer membrane (PVDF Polyscreen, NEN Life Science Products, Boston, MA, USA) using a semi-dry blotting device (Trans-blot SD, BioRad, München, Germany). Immunodetection was carried out by using affinity-purified monoclonal anti-GFP antibody JL-8 (Living Colors, Clontech, Palo Alto, U.S.A) and chemiluminescence as described in [26].

### Cell cycle analysis

For cell cycle analysis, prostate cells were trypsinized and washed with ice-cold PBS. Then cells were fixed with 70% ice-cold ethanol for 1 hour, followed by incubation in freshly prepared nuclei staining buffer (200 μg/ml RNase plus 20 μg/ml Propidium Iodide-PI in PBS) for 1 hour at 37°C. Cell cycle histograms were generated after analysis of PI-stained cells by fluorescence-activated cell sorting (FACS) with a Becton Dickinson FACVantage SE Cell Sorter. For each sample, triplicates were performed and >1 × 10^4 ^events were recorded. Histograms generated by FACS were analyzed by Cell Quest Software to determine the percentage of cells in each phase (G1-S-G2/M). Statistical evaluation of the data was done by paired Student's t test using the SPSS 11.5 software for Windows. SE and P values are shown where appropriate.

### Reverse transcription polymerase chain reaction (RT-PCR)

Total RNA was isolated by using the RNeasy Mini kit (Qiagen GmBH, Hilden, Germany), following the manufacturer's recommendations. RNA (2.5 μg) of PC3 and DU145 cells were converted to cDNA by SuperScript III Reverse Transcriptase, using a mixture of Oligo dT (0.25 μg) and Random Primer (0.5 μg; all from Invitrogen, Carlsbad, CA, USA) in a volume of 11 μl RNase-free water. Gene expression (Table 1) was quantified in triplicate with the iCycler and MyIQ amplifiers (Bio-Rad) using a SYBR-Green Supermix kit as recommended by the supplier (Bio-Rad). The real-time PCR was performed with primers and annealing temperatures described in table 2; expression of each of the *INTS6/DICE1 *target genes was quantified in three distinct batches of transfection.

**Table 2:** Genes and corresponding primer sequences

Gene Symbol	Forward Primer 5' → 3'	Reverse Primer 5' → 3'	Amplicon size (bp)	Annealing Temp. (°C)
G3PDH	TGGTATCGTGGAAGGACTCA	ATGCCAGTGAGCTTCCCGTT	188	58
DICE1	TGCCCATCTTACTGTTCCTG	TCTTCGAAAGTGACCAGC	169	58
CXXC4	TGCAAGAGGCTCATCAACTG	TCATTTCCAAATGCCTTGAA	204	60
CCND1	AGGAACAGAAGTGCGAGGAG	GGCGGATTGGAAATGAACT	394	60
TCF7L1	ACGAGCTGATCCCCTTCC	TGACCTCGTGTCCTTGACTG	400	60

### RT^2 ^Profiler PCR array system

The expression of 84 genes related to human Wnt signaling pathway was analyzed in PC3 cells by quantitative real-time PCR using RT^2 ^Profiler PCR Array technology (SABiosciences, Frederick, MD, USA). The provided Master Mix, containing dNTP's, polymerase, MgCl_2 _and SYBR-Green, and the cDNA were used as described by the manufacturer. The RT-PCR reaction was performed in the iCycler (Bio-Rad). For data analysis Ct values were used for the gene of interest and the housekeeping gene glyceraldehyde-3-phosphate dehydrogenase (*G3PDH*). The relative quantification of gene expression was determined using the 2^-ΔΔC^T method as described by Livak and Schmittgen [27].

## Competing interests

The authors declare that they have no competing interests.

## Authors' contributions

SF performed transfection, colony formation, apoptosis and necrosis assays, analyzed data and wrote the manuscript. JH and AW performed RT-PCR analysis, MS performed Western blot analysis, CS performed Northern blot analysis, MHS and SF performed cell cycle analysis, TN analyzed data and wrote the manuscript, IW constructed plasmids, conceived the project, analyzed data and wrote the manuscript. All authors read and approved the manuscript to be submitted for publication.
